# Alternative Treatment of the Resistant-to-Treatment Tourette Syndrome—A Systematic Review

**DOI:** 10.3390/jcm15093393

**Published:** 2026-04-29

**Authors:** Agata Czech, Magdalena Czarnecka, Olga Grodzka, Piotr Chądzyński, Izabela Domitrz

**Affiliations:** 1 Department of Neurology, Faculty of Medicine and Dentistry, Medical University of Warsaw, Ceglowska 80 Street, 01-809 Warsaw, Poland; s091897@student.wum.edu.pl (A.C.); s090679@student.wum.edu.pl (M.C.);; 2 Doctoral School, Medical University of Warsaw, Żwirki i Wigury 61 Street, 02-091 Warsaw, Poland

**Keywords:** alternative treatment, alternative therapy, neurodevelopmental disorders, non-pharmacological approaches, refractory Tourette syndrome, tics

## Abstract

**Background:** Tourette syndrome (TS) is a chronic neurodevelopmental disorder with a significant rate of patients remaining refractory to standard treatments. Refractory TS is defined as the persistence of clinically significant tics causing functional impairment despite adequate trials of both behavioral therapy and at least two generally accepted pharmacological treatments from different classes, administered at appropriate doses and durations. This systematic review synthesizes the current evidence on alternative pharmacological and non-pharmacological interventions for treatment-resistant TS. **Methods:** The review was conducted in accordance with the Preferred Reporting Items for Systematic Reviews and Meta-Analyses (PRISMA) 2020 guidelines. PubMed, Embase, and Scopus were searched for studies published from 2010 onward. Eligible publications included randomized and non-randomized clinical studies, and case reports evaluating alternative interventions in refractory TS, excluding deep brain stimulation. The primary outcome measures included validated scales assessing tic severity and functional impairment, with available data demonstrating reductions in tic severity, including clinically meaningful improvements reported in several studies. Thirteen studies met the inclusion criteria, comprising five clinical studies (including three randomized trials) and eight case reports. Heterogeneity across studies was primarily driven by differences in study design, patient populations, and variability in intervention types, protocols, and outcome assessments. **Results:** Investigated interventions included cannabinoids, valproic acid, deep transcranial magnetic stimulation, neurofeedback, biofeedback, electroconvulsive therapy, and ablative neurosurgical procedures. Cannabinoid-based treatments showed potential reductions in tic severity; however, results were inconsistent and often not statistically significant, with evidence largely derived from small or uncontrolled studies. Evidence for non-pharmacological approaches was limited and largely derived from individual cases, with some modalities showing potential benefit in specific subgroups, such as patients with comorbid obsessive-compulsive disorder. **Conclusions:** Overall, alternative interventions may offer therapeutic value for selected individuals with treatment-resistant TS, with the greatest data regarding cannabinoid use; however, the current evidence base remains heterogeneous and methodologically constrained. Deep transcranial magnetic stimulation might be beneficial in a specific patient population. Larger, well-controlled studies are required to clarify efficacy, safety, and treatment response.

## 1. Introduction

Tourette syndrome (TS) constitutes a chronic neurodevelopmental disorder with onset in childhood, characterized by the presence of both motor and vocal tics. Diagnostic criteria established by the European Society for the Study of Tourette Syndrome (ESSTS) require motor, vocal, or combined tics to persist for at least 12 months and not be attributable to another medical condition or medication [1]. According to independent sources, the age of symptom onset should occur either before age 21, under the ESSTS guidance, or before age 18, according to the Diagnostic and Statistical Manual of Mental Disorders, Fifth Edition [DSM-5] [1,2,3]. TS is more prevalent in males than females (approximately 4:1) [4], and the clinical course is often reported as more severe in males. The pathophysiology of TS remains incompletely understood. Converging evidence implicates genetic, immunological, and environmental contributions as potential risk factors [5]. A leading hypothesis posits a dysfunction within corticobasal ganglia–thalamocortical circuits, resulting in impaired striatal inhibition; however, this account does not fully explain cardinal features of tics, including their waxing–waning course and premonitory urges [4]. Alternative models propose abnormally increased, phasic dopaminergic release that reinforces persistent motor habits and induces maladaptive neuroplastic changes [4,6]. Electroencephalography (EEG)-based analyses further suggest that tics may represent responses to internal or external stimuli [4]. Furthermore, comorbid conditions, particularly obsessive-compulsive disorder (OCD) and attention-deficit/hyperactivity disorder (ADHD), are common in TS [4], which may represent a relevant factor to explore in further analyses of pathogenetic mechanisms. Tic severity is variable over time, typically exacerbating under stress or fatigue and improving with focused attention. A subset of individuals can voluntarily suppress tics for limited periods [2]. Although symptoms may attenuate with age for some, many adults continue to experience impaired quality of life [2]. Behavioral therapy is considered a first-line treatment, aiming to enhance voluntary control over tics and addresses maintaining or exacerbating factors [7]. When response is insufficient, pharmacotherapy is commonly introduced, using agents such as haloperidol, aripiprazole, or clonidine [8]. Among the dopaminergic-modulating treatments, aripiprazole is often highlighted for a comparatively favorable adverse-effect profile relative to other antipsychotic drugs used in the treatment of TS [8]. Nevertheless, a proportion of patients exhibit inadequate response or discontinue treatment due to adverse effects, underscoring the need for alternative approaches.

Emerging and adjunctive interventions described in the literature include deep transcranial magnetic stimulation (deep TMS), electroconvulsive therapy (ECT), neurofeedback and biofeedback methods, ablative procedures such as capsulotomy, and alternative pharmacologic options including valproic acid and cannabinoids. While some modalities have shown promise in reducing tic severity, their efficacy and safety profiles remain insufficiently established. Therefore, this systematic review aimed to synthesize evidence on pharmacologic and non-pharmacologic interventions for treatment-resistant TS. Specifically, it evaluates the efficacy, tolerability, and clinical utility of deep TMS, neurofeedback, biofeedback, ECT, capsulotomy, and the use of valproic acid and cannabinoids as representative examples of emerging or less commonly applied therapeutic strategies.

## 2. Methodology

The systematic review was conducted in accordance with the Preferred Reporting Items for Systematic Reviews and Meta-Analyses (PRISMA 2020) guidelines [9]. The completed PRISMA checklist is provided in the Supplementary Materials (Table S4). Three databases were searched: PubMed Database, Embase Database, and Scopus Database, using a similar search strategy. The search strategy was adapted to each database using both controlled vocabulary (e.g., MeSH terms in PubMed and Emtree in Embase) and free-text keywords. Boolean operators and database-specific filters were applied where appropriate. A detailed search strategy is provided in Table S1. The systematic review was registered in PROSPERO (ID of the review protocol CRD420251007338). The review protocol was followed throughout the study without significant deviations.

### 2.1. Inclusion and Exclusion Criteria

The review aimed to analyze alternative treatment methods in the management of refractory-to-treatment TS. Therefore, studies included had to cover this specific issue. Research focusing on conditions other than TS or generally approved therapies in TS was excluded. Moreover, studies investigating deep-brain stimulation (DBS) in TS were not included in this review. This decision was based on the fact that DBS represents a well-established and extensively studied therapeutic modality, supported by a substantial body of literature, including multiple systematic reviews and meta-analyses. In contrast, the present review was specifically designed to focus on alternative and less well-characterized interventions, for which the available evidence remains limited, heterogeneous, or emerging. By narrowing the scope in this way, we aimed to provide a more targeted synthesis of therapeutic approaches that are currently less clearly defined in the literature and may benefit from further investigation. Considered studies included randomized clinical trials, observational studies (such as case-control, cohort, and cross-sectional studies), case series, and case reports. We excluded the following article types: reviews (either narrative, scoping, or systematic), meta-analyses, commentaries, and conference abstracts. Grey literature and unpublished data were not systematically included in this review, as the analysis was limited to peer-reviewed and publicly available sources to ensure data reliability and consistency. This approach may introduce a risk of publication bias. However, clinical trial registries were screened to identify relevant studies, and where results were available in published form, these were incorporated into the analysis. Trial registry records were also cited where appropriate. Furthermore, we included studies written in English, since this language is one of the authors’ languages of fluency and allows understanding by a wide range of readers; articles in other languages were not considered. Finally, since our topic regards a fast-developing issue, we included only articles from 2010 or later to keep the review up-to-date and omit out-of-date studies.

### 2.2. The Selection Process

Three databases were screened to find all eligible studies for the review: the PubMed Database (134 records), the Embase Database (86 records), and the Scopus Database (56 records). The selection process was performed by two independent reviewers (A.C. and M.C.), who categorized reports into the following groups: duplicates, excluded by title or type, excluded by abstract, excluded by full text, and included. Any discrepancies were subsequently analyzed by the third author (O.G.) and resolved through discussion. Out of the 276 records analyzed, 24 were excluded based on the year of publication, and 6 were removed as duplicates identified across databases. Thus, 246 articles were screened, and 203 were removed by title or type. Further abstract analysis led to the exclusion of 20 studies. Finally, ten studies were removed based on full-text screening. Therefore, 13 eligible articles were included in the systematic review. No studies that appeared to meet the inclusion criteria were excluded after full-text assessment.

**Figure 1:**
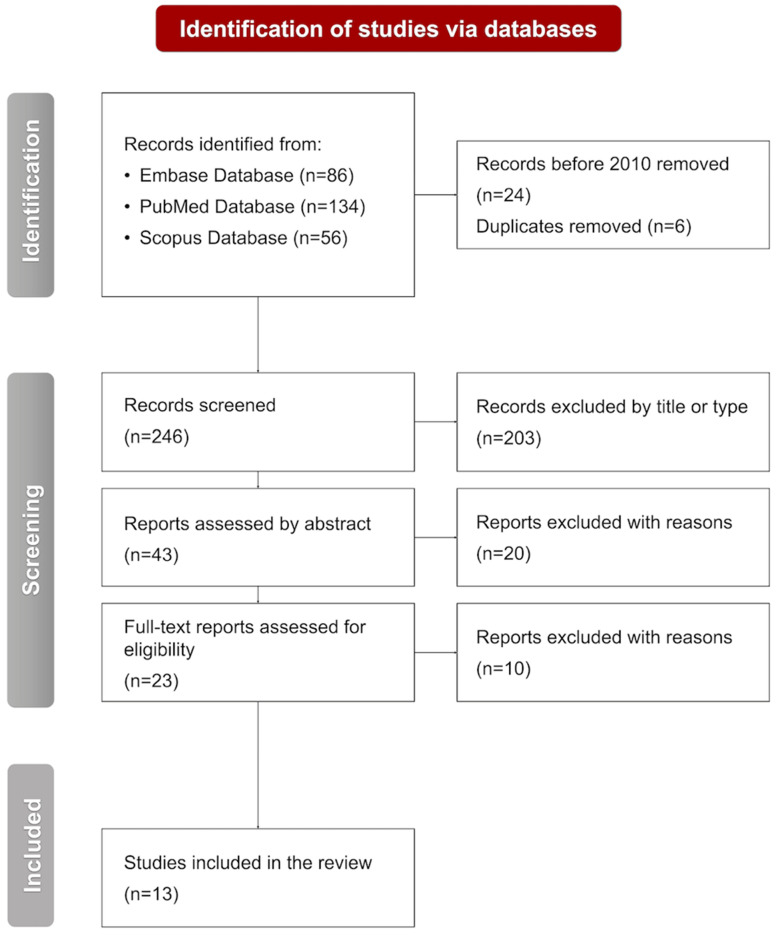
A flowchart presenting the selection process performed in accordance with the PRISMA 2020 guidelines. n, number of studies.

The selection of interventions was guided by the availability of published evidence and their emerging clinical relevance and was not intended to represent an exhaustive overview of all possible therapeutic options for TS. The selection process is presented in Figure 1.

### 2.3. Data Curation and Synthesis

Data curation was conducted independently by two reviewers (A.C., M.C.), who extracted relevant information from the included studies using a predefined framework aligned with the study objectives. Extracted data included study characteristics, patient populations, treatment modalities, and reported outcomes. Discrepancies between reviewers were resolved through discussion and consensus.

The synthesis of data was performed using a structured approach. Studies were grouped according to treatment type, and findings were systematically compared to identify patterns, consistencies, and differences across the included literature. This approach enabled an integrated and comprehensive interpretation of the available evidence.

The primary outcomes of interest included the effectiveness of alternative treatment approaches in TS, assessed in terms of tic severity, frequency of tics, and quality of life. Tic severity was defined as the overall intensity and clinical burden of motor and vocal tics, while tic frequency referred to the number or occurrence of tics over a specified period. Quality of life was evaluated as the impact of symptoms on daily functioning, psychological well-being, and social participation. Additional variables extracted from the included studies comprised participant characteristics (e.g., age, sex, sample size), study design, and details of the interventions, including type and duration. Effect measures included mean differences or standardized mean differences in disease severity scores, depending on the measurement scales used across studies; however, these were used for descriptive comparison only. The synthesis should therefore be interpreted as primarily descriptive and exploratory, reflecting the limited methodological quality of the available evidence. Due to variability in outcome reporting, emphasis was placed on the Yale Global Tic Severity Scale–Total Tic Score (YGTSS-TTS), where available, to improve comparability across studies.

Given the heterogeneity of the included studies in terms of study design, patient populations, and intervention characteristics, a formal quantitative assessment of heterogeneity was not performed. Instead, potential sources of heterogeneity were explored qualitatively through the subgrouping of studies based on intervention type and comparison of findings across different study settings and populations. Although standardized effect measures were extracted where available, a formal meta-analysis was not conducted due to substantial heterogeneity in study design, populations, interventions, and outcome reporting.

### 2.4. Risk-of-Bias Assessment

Risk-of-bias assessment was conducted according to the study design. Randomized controlled trials were evaluated using the Cochrane Risk of Bias 2 (RoB 2) tool, assessing domains such as the randomization process, deviations from intended interventions, missing outcome data, measurement of the outcome, and selection of the reported result. Case reports and case series were assessed using the Joanna Briggs Institute (JBI) critical appraisal tools, considering factors such as patient selection, reporting clarity, intervention description, and outcome assessment. Due to heterogeneity, the results were summarized descriptively. The detailed results of the risk-of-bias assessment are provided in Tables S2 and S3.

## 3. Results

The systematic search yielded thirteen eligible publications: three randomized controlled trials, two non-randomized interventional studies, and eight case reports. These investigations addressed alternative interventions for treatment-resistant TS and encompassed both pharmacologic (number (*n*) = 8) and non-pharmacologic (*n* = 5) approaches. The intervention spectrum comprised cannabinoids, valproic acid, deep TMS, neurofeedback, biofeedback, ECT, and neurosurgical procedures (including stereotactic thermocoagulation). Sample sizes ranged from single-patient reports to clinical trials enrolling approximately 100 participants. The results of the risk-of-bias assessment are presented in Tables S2 and S3.

### 3.1. Pharmacological Interventions for Refractory Tourette Syndrome

Seven publications [10,11,12,13,14,15,16] evaluated Δ9-tetrahydrocannabinol (THC)-based preparations: pure THC, THC/cannabidiol (CBD) combinations (nabiximols; Sativex^®^), and THC co-administered with palmitoylethanolamide (PEA), and one addressed valproic acid. The cannabinoid corpus comprised five case reports (four single-case [12,14,15,16] and one two-patient report [13]), one Phase II pilot study (*n* = 16) [11], and one randomized, multicentered Phase IIIb trial (*n* = 97) [10], yielding a total of 119 participants across pharmacologic studies.

The protocol of the CANNA-TICS study was indicated as a relevant study to include in the review on the basis of its high scientific relevance [17]; thus, the publication of corresponding results, identified through supplementary targeted searching, is reported [10]. This trial reported that a ≥25% reduction in YGTSS-TTS was achieved by 21.9% of patients receiving nabiximols versus 9.1% with the placebo (*p* = 0.07). To facilitate understanding of the included studies, it is worth mentioning that the YGTSS is currently the primary scale for determining the severity of TS symptoms. The scale consists of two categories: an assessment of the severity of motor and vocal tics, in which a total of 50 points can be obtained, known as the Total Tic Score, and the overall impact of tics on functioning (the Impairment Score), which also has a maximum score of 50 points, giving a total of 100 points. Coming back to the currently described study and given that the *p*-value was close to significance, the research indicated a potentially clinically meaningful, though statistically non-significant, advantage. A tendency to be significant highlights the need for further studies comprising a larger group of patients, which, undoubtedly, requires more attention on this highly specific but relevant topic. In patients with comorbid ADHD, nabiximols was associated with a greater reduction in YGTSS-TTS compared with the placebo, showing a large effect that did not reach conventional statistical significance and should therefore be interpreted with caution in this secondary analysis. No detrimental effects on driving performance were detected. Adverse events occurred in 95.3% of nabiximols-treated participants versus 78.8% on the placebo (*p* = 0.003); however, they were predominantly mild (e.g., somnolence, xerostomia, dizziness).

In a Phase II pilot study (Bloch et al., 2021) testing THC + PEA, the mean YGTSS-TTS improvement was the reduction of 7.9 points from the baseline (*p* = 0.002) [11]. All participants reported mild adverse effects (somnolence, fatigue) that were mitigated by evening dosing and/or dose adjustment. The summary of clinical studies analyzed in this paragraph is presented in Table 1.

Furthermore, all five case reports documented clinical improvement. In a study by Hasan et al., a 15-year-old experienced a YGTSS decrease from 97 to 54 after seven weeks of delta-9-THC [12]. A paired-pulse TMS demonstrated increased short-interval intracortical inhibition (SICI, 75%→85%) and a prolonged cortical silent period four hours post-dose. Jakubovski and Müller-Vahl described two patients with severe vocal tics, a 19-year-old treated pharmacologically since age 9 and a 16-year-old treated since age 14, who showed improvement on medical cannabis and dronabinol, respectively, with subjectively assessed gains in speech fluency [13]. Pichler et al. reported a 40-year-old woman with short-lived benefit from self-grown cannabis who subsequently achieved a sustained YGTSS reduction from 73 to 44 after two months on a standardized 1:2 THC:CBD tincture; however, xerostomia was noted [14]. Kanaan et al. described a 22-year-old patient with severe complex vocal tics (including pronounced coprolalia, echolalia, and spitting) and severe complex motor tics, who showed a 22.2% reduction in YGTSS-TTS on nabiximols (45→35), marked quality-of-life improvement on the Gilles de la Tourette Syndrome–Quality of Life Scale (GTS-QOL) (51→11), and a 35.3% improvement on the Premonitory Urge for Tics Scale (PUTS) (34→22) [15]. Both scales are subjective assessment tools used to evaluate the patient’s condition. The GTS-QOL is a self-report questionnaire consisting of 27 questions that assess quality of life across four categories: psychological, physical, cognitive, and social. In contrast, the PUTS measures patients’ subjective sensations associated with the urge to perform a tic, based on 9 or 10 questionnaire items. Here, it is worth mentioning that the previously described CANNA-TICS study did not detect between-group differences on PUTS (PUTS-9 *p* = 0.21; PUTS-10 *p* = 0.28). Finally, in a publication by Trainor et al., a 26-year-old patient, being a refractory case of TS treated with Sativex^®^ (THC:CBD 1:1) improved from YGTSS 71 to 46 within four weeks and showed large reductions in the Original Rush Videotape Rating Scale (motor tics 176→27, −85%; vocal tics 20→2, −90%) [16].

Moving to another type of alternative pharmacological treatment, Ye and Lippmann detailed a 10-year-old with TS and bipolar disorder who improved on sodium valproate (YGTSS 80→24), with benefit sustained for over two years [18]. The authors posited the modulation of the gamma-aminobutyric acid (GABA) system as a plausible mechanism and emphasized standard safety considerations, since valproic acid is known to cause significant adverse effects, including hepatotoxicity or hematologic abnormalities, and should be implemented with caution.

Across heterogeneous designs, cannabinoid-based interventions generally trended toward tic reduction, with the strongest evidence base for nabiximols and THC-containing regimens; tolerability was acceptable but characterized by frequent, mostly mild adverse events. Single-case evidence suggests potential benefits of valproate in select comorbid presentations, warranting careful consideration given known risks. Robust, adequately powered trials with standardized outcome frameworks (e.g., YGTSS-TTS and impairment, PUTS, quality-of-life measures) are needed to clarify efficacy, safety, and patient-level moderators of response.

### 3.2. Non-Pharmacological Methods of Treating Refractory Tourette Syndrome

In this section, we describe five publications [19,20,21,22,23] that examine non-pharmacological approaches to the treatment of refractory TS. Two of the included publications were case reports [22,23], one employing neurofeedback combined with imagery training [23], and one evaluating ECT [22]. The remaining three were clinical trials [19,20,21], including one randomized study of biofeedback (*n* = 21) [20]. The two non-randomized trials assessed repetitive/deep TMS (*n* = 12) [21] and stereotactic thermocoagulation (*n* = 50) [19]. In total, these reports encompassed 86 participants.

Stereotactic thermocoagulation is a very precise neurosurgical intervention. In cases of TS, the potential targets for this procedure are the anterior or posterior one-third of the anterior limb of the internal capsule, where a radiofrequency current can be applied to create a focal thermolesion at 60–80 °C. This method was detailed by Yu-Hui Li et al., who enrolled 52 patients (31 males, 21 females) aged 13–32 years in their study from whom thirty-eight met the criteria for TS, and twelve were diagnosed with persistent motor and/or vocal tic disorders [19]. At 6 months, mean YGTSS reductions were 8.8 points (approximately 40%) for motor tics and 5 points for vocal tics, corresponding to an overall improvement of 28 points (approximately 35%). At 12 months, the total improvement was sustained at 24 points (approximately 29%). Notably, motor tics improved to a greater extent than vocal tics.

The second-largest study was a randomized clinical trial by Nagai et al. evaluating biofeedback, which provides real-time visual or auditory feedback to facilitate voluntary modulation of physiological responses [20]. Twenty-one participants (mean age 26.7 ± 10.6 years) were scheduled for twelve 30-minute sessions over four weeks; nine individuals (six in the active arm and three in the control arm) discontinued, most frequently citing low motivation. The between-group difference in change in electrodermal activity did not reach statistical significance. Across training, neither group demonstrated improved voluntary control of electrodermal activity, an index of autonomic arousal regulation, and this lack of physiological change did not translate into a reduction in tic severity. No statistically significant between-group differences were observed for motor or vocal tics.

Another clinical study, this one examining the effectiveness of deep TMS, was performed by Bloch et al. [21]. Twelve patients aged 18–65 years were enrolled, of whom two discontinued the 4-week protocol of 20 sessions due to insufficient perceived benefit. Participants received inhibitory stimulation of the medial premotor area with brief, repeated magnetic pulses at 1 Hertz (Hz) for 5 min with 2-minute inter-train intervals. Half of the sample had comorbid OCD. Reported adverse effects were mild (scalp discomfort in two participants; headache in three). Greater improvement in tic severity, reaching clinical significance, was observed among individuals with comorbid OCD, accompanied by improvement in OCD symptoms, whereas outcomes among participants with TS alone were not statistically significant. Importantly, the correlation between changes in OCD severity and tic severity was insignificant, suggesting potentially independent effects of the intervention on these symptom domains. The summary of clinical studies analyzed in this paragraph is presented in Table 2.

In a case report by Dehning et al., the impact of ECT on tic reduction was assessed in a 35-year-old man with TS and comorbid OCD [22]. The patient underwent an acute course of 14 ECT sessions at 50 Hz, followed by maintenance therapy: monthly sessions for 10 months, then five sessions at 6-week intervals, and subsequently three sessions at semiannual intervals. Thereafter, a total of 23 additional maintenance sessions were administered. Over the 5-year observation period, the YGTSS scores declined from 50 to 0.

Lastly, in the case report by Zhuo and Li, two patients aged 14 and 16 received neurofeedback combined with imagery training [23]. The authors suggest that the combined protocol yielded superior symptom relief. Each patient completed 80 neurofeedback sessions. In the 16-year-old, the YGTSS decreased from 87 to 50 points, with only occasional hand tics remaining; no adverse effects were noted. In the 14-year-old, improvement was observed, with a reduction from 73 to 40 YGTSS points. Follow-ups at three, six, and twelve months confirmed maintenance of therapeutic benefit, and no significant adverse events were reported.

## 4. Discussion

**Figure 2:**
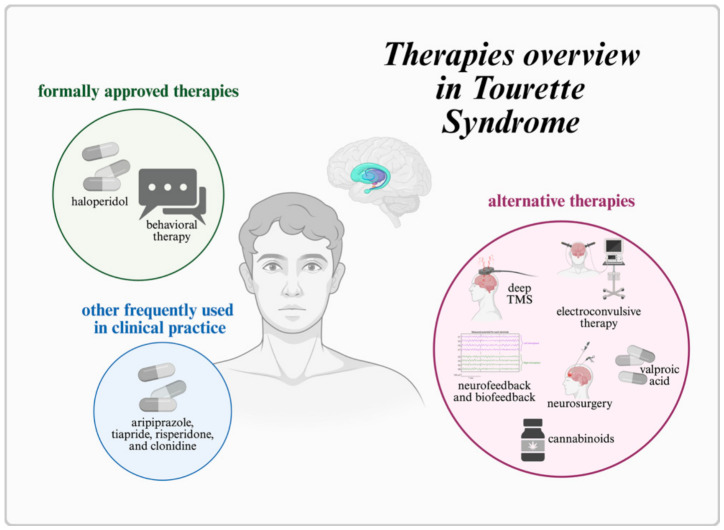
A simplified graphical overview of selected therapies currently approved or used in the treatment of Tourette syndrome, as well as representative alternative approaches. The figure is intended for illustrative purposes only and does not reflect the complexity of treatment pathways or provide a comparative assessment of therapeutic efficacy. TMS, transcranial magnetic stimulation.

Currently, haloperidol remains the only medication formally approved in Europe for the treatment of TS. Other agents, such as aripiprazole, tiapride, risperidone, and clonidine, are used in clinical practice but lack a specific marketing authorization for TS, underscoring the limited scope of pharmacotherapy [8]. In the treatment of TS, behavioral therapy is recommended as the first-line intervention, and pharmacological treatment is considered when behavioral approaches are insufficient or unavailable [8]. Pharmacotherapy is individualized according to the patient’s needs and may be adjusted or switched to alternative antipsychotic medications in cases of inadequate efficacy or poor tolerability [8]. A critical challenge arises when the expected benefits are not realized despite the use of all approved or commonly recommended treatments. Up to approximately 30% of patients exhibit an unsatisfactory response or experience adverse effects severe enough to preclude continuation [24]. This treatment-resistant subgroup highlights the need to identify and evaluate alternative therapeutic strategies. Available treatment options with proposed alternative therapies are presented in Figure 2. Importantly, the overall strength of evidence remains limited. Most included studies were small, non-randomized, or case-based, which increases susceptibility to bias, placebo effects, and regression to the mean. Therefore, the findings should be interpreted as hypothesis-generating rather than conclusive. Greater interpretative weight should be assigned to findings from randomized controlled trials compared to observational studies and case reports.

One proposed approach for refractory TS is the use of cannabinoids. The endocannabinoid system plays a key role in regulating central nervous system functions, with cannabinoid receptor type 1 (CB1) receptors abundantly expressed in basal ganglia structure including the caudate nucleus and striatum, which are involved in motor control [25]. Preclinical studies in murine models have shown that CB1 receptor antagonism (e.g., with rimonabant) can exacerbate tic-like behaviors, cognitive deficits, and hyperactivity, effects reported as particularly pronounced in males [26]. These findings suggest that mechanisms counteracting CB1 signaling may contribute to TS pathogenesis.

A principal barrier to regulatory approval and broad implementation of cannabinoids in refractory TS is the paucity of well-designed clinical trials with rigorous methodology. In our review, seven publications assessed cannabinoids in treatment-resistant TS, yet only one was a randomized controlled trial. The remainder comprised case reports or uncontrolled designs, substantially limiting evidentiary strength and necessitating caution in interpretation. Additional adequately powered, high-quality trials are urgently needed. As shown in Tables S2 and S3, most included studies were assessed as having a high risk of bias, which substantially limits the strength of conclusions.

The included studies evaluated a range of preparations. Composite formulations merit attention given the putative “entourage effect”, whereby co-constituents may potentiate THC’s therapeutic actions by modulating metabolism or receptor availability [27]. This could yield improved efficacy at lower doses and thereby mitigate dose-related adverse effects. Although conceptually compelling, this hypothesis requires confirmation in trials directly comparing THC alone with THC + CBD or THC + PEA. Reported adverse effects were generally mild, most commonly somnolence, xerostomia, fatigue, euphoria, and headache, and, in one study, were attenuated by dose adjustments or evening administration. Nevertheless, inappropriate use or misuse of cannabinoids can lead to dependence and other harms, which may constrain their clinical utility [28].

Interpretation of cannabinoid studies should also consider deliberate tic suppression during assessments and the influence of placebo responses. A proportion of individuals with TS can partially or temporarily suppress tics, which complicates outcome evaluation and may inflate apparent treatment effects [4], particularly when reliance on subjective scales such as the YGTSS is high. This concern is consistent with findings from the CANNA-TICS trial, in which the active arm did not achieve significantly greater improvement than the placebo. These observations reinforce the need for more randomized, blinded designs in which participants are unaware of treatment allocation.

Another important issue is the lack of standardization across studies, both in the formulations used (THC form, carrier, dose, and treatment duration) and in outcome measurement (e.g., use of different YGTSS versions; omission of PUTS or GTS-QOL in some studies). Such heterogeneity precludes direct comparisons and hinders definitive conclusions. Despite these limitations, some studies reported reductions in tic severity; however, these findings were inconsistent and primarily derived from small or uncontrolled studies, suggesting a potential signal of efficacy that requires confirmation in larger, well-controlled trials. Notably, the only adequately powered randomized controlled trial by Müller-Vahl et al. did not demonstrate statistically significant efficacy, while the study by Bloch et al. lacked a placebo control, further limiting interpretability. Overall, these findings should be interpreted with caution, and clinical decision-making should remain individualized, taking into account the patient’s symptom profile, comorbidities, preferences, and risk tolerance.

Among the non-pharmacological modalities, deep TMS is notable as a non-invasive alternative to DBS. In the study included in this review, patients with TS comorbid with OCD achieved statistically significant improvements in tic severity compared with those without OCD. This pattern is consistent with the possibility of distinct pathophysiological mechanisms across TS subtypes defined by comorbidity profiles. Neuroimaging evidence indicates that TS co-occurring with OCD engages in neural networks that differ from those implicated in TS alone [29], which may help identify patients most likely to benefit from deep TMS and require further investigations. The safety profile was favorable, with no serious adverse events reported, suggesting that deep TMS may represent a potential option in selected patients with TS and comorbid OCD, who are limited by adverse effects to other therapies. It is noteworthy, however, that the protocol required 20 stimulation sessions, implying that intensive, repeated treatment may be necessary for clinical benefit, an important practical and economic consideration for patients with limited capacity to attend frequent sessions.

A related principle, modulation of cortical activity, underpins ECT. Based on the limited evidence included in this review, it may be hypothesized that although ECT and deep TMS differ markedly in invasiveness and scope of neural effects, both may induce functional changes in networks implicated in tic generation. In the included case report, remission of tics and self-injurious behaviors was achieved within six weeks of initiating ECT, with maintenance therapy sustaining benefit over several years. Despite its rapid potential efficacy, ECT remains controversial and raises ethical considerations. While no major adverse effects were reported in the case described, prolonged treatment may plausibly carry risks such as cognitive impairment [30]. ECT should therefore be reserved for exceptional circumstances, particularly severe clinical presentations unresponsive to, or contraindicated for, other interventions.

Neurofeedback and biofeedback comprise biologically based feedback methods designed to facilitate volitional control over selected physiological processes. Their advantages include non-invasiveness, a minimal adverse-effect profile, and applicability in patients who cannot tolerate medications or are ineligible for interventional treatments. In some patients treated with neurofeedback, clinical improvements persisted beyond the active intervention. Augmenting neurofeedback with imagery training may enhance engagement and neuroplastic change, potentially amplifying therapeutic effects. Practical limitations include the time-intensive nature of treatment; benefits were observed after dozens of sessions, which can pose logistical and motivational barriers. Moreover, the absence of standardized protocols complicates cross-study comparisons and the development of best-practice guidelines.

In contrast, the randomized biofeedback study targeting sympathetic electrodermal activity did not demonstrate clear efficacy for tic reduction. Although transient modulation of physiological arousal was achievable, this did not translate into durable symptomatic improvement. Whether alternative biofeedback targets (e.g., electromyography or heart-rate variability) or longer, more intensive protocols might yield superior outcomes remains an open question. Based on current evidence, biofeedback, at least in the evaluated form, appears to have limited therapeutic value in TS. A shared constraint of both neurofeedback and biofeedback is the requirement for sustained, active patient engagement, which may limit feasibility in individuals with attentional difficulties or substantial comorbid psychopathology.

The only surgical approach included in this review was stereotactic thermocoagulation of the anterior limb of the internal capsule, an anatomic target also used in neurosurgical treatment of severe OCD [31]. As noted, some interventions yielded more pronounced benefits in patients with comorbid OCD, suggesting shared pathophysiological mechanisms across the disorders. The analyzed study reported significant post-procedural improvements in tics and associated behavioral symptoms, particularly when lesions targeted the posterior portion of the anterior limb of the internal capsule. Although the method may be highly effective, with a relatively rapid onset of benefit in a meaningful subset of patients, the procedure carries a risk of neuropsychiatric complications, the severity of which can be difficult to predict. The most common include frontal syndrome, as well as somatic effects such as headache, ataxia, and transient urinary incontinence. Less frequent but more severe complications include hypomania/mania, suicidal ideation, cognitive deficits, ischemic infarcts affecting deep brain structures, and hemiplegia [32]. Given its irreversible nature and the limited number of controlled studies, capsulotomy should be considered only for individuals with severe, drug-resistant TS after exhaustive evaluation of less invasive alternatives. The strongest evidence currently derives from a limited number of randomized controlled trials, while the majority of findings are based on lower-level evidence. Future studies should focus on well-powered randomized controlled trials using standardized outcome measures such as YGTSS, PUTS, and quality-of-life scales.

Beyond efficacy, several practical and clinical considerations should be considered when evaluating alternative treatments for TS. These include long-term safety, cost-effectiveness, accessibility, and regulatory status, which remain insufficiently addressed in the current literature. Many of the interventions discussed are associated with limited long-term data, variable availability across clinical settings, or substantial logistical and financial burdens. Additionally, the lack of formal regulatory approval for most approaches further complicates their integration into routine clinical practice. These factors should be considered alongside efficacy outcomes when interpreting the potential clinical utility of these therapies.

### Limitations

Methodological and implementation limitations of the included studies are addressed in Section 4. In addition, the present review has constraints related to its own methodology. We screened three databases, which do not encompass all potential sources and therefore carry a risk of missing relevant studies. Nevertheless, querying more than one database likely mitigated, though did not eliminate, this risk. Furthermore, the exclusion of grey literature and restriction to English-language publications may have introduced publication bias and limited the comprehensiveness of the evidence base. This approach was adopted to ensure accessibility and consistency of the included studies for an international readership. 

## 5. Conclusions and Future Directions

The pharmacological and non-pharmacological interventions reviewed here show therapeutic promise for refractory TS, yet each is constrained by important limitations. Most notable are the small number of rigorously designed clinical trials, the paucity of data on long-term effectiveness, and the incomplete characterization of safety and tolerability. At the same time, frequent comorbidity with OCD and the comparatively more favorable responses observed in TS patients with comorbid OCD suggest the value of further investigation into shared pathophysiological mechanisms and their therapeutic implications. Given the relative underrepresentation of TS in the neurological research landscape, sustained efforts to develop, standardize, and robustly evaluate TS treatments remain a priority.

## Figures and Tables

**Table 1:** A summary of clinical studies regarding the use of cannabinoids in refractory Tourette syndrome.

Ref.	Year	Study Type	Population	Comparison	TS Severity Scale	Results
Muller-Vahl et al. [10]	2023	Prospective, multicenter, randomized, double-blind, placebo-controlled, Phase IIIb superiority trial	64 adult pts with CTD, baseline YGTSS-TTS ≥ 14 for TS or ≥10 for CMT/CVT, and CGI-S ≥ 4	33 pts with CTD receiving placebo	YGTSS-TTS	≥25% reduction in YGTSS-TTS in 21.9% of patients treated with nabiximols vs. 9.1% with placebo (*p* = 0.07); greater tic reduction in men and in patients with comorbid ADHD.
Bloch et al. [11]	2021	Phase II uncontrolled interventional trial	16 adult pts with TS (10 M, 6 F)	No control group	YGTSS-TTS	7-point reduction in total YGTSS-TTS at 12 weeks (≈20%); improvement observed within 1 week

ADHD, attention-deficit/hyperactivity disorder; CMT, chronic motor tic; CTD, chronic tic disorder; CVT, chronic vocal tic; F, females; CGI-S, Clinical Global Impression-Severity; M, males; pts, patients; Ref., reference; TS, Tourette syndrome; YGTSS-TTS, Yale Global Tic Severity Scale-Total Tic Score.

**Table 2:** A summary of clinical studies regarding non-pharmacological approaches in refractory Tourette syndrome.

Ref.	Year	Study Type	Population	Comparison	TS Severity Scale	Results
Li et al. [19]	2020	Prospective clinical case series	52 pts (38 TS, 12 persistent tic disorder; 31 M, 21 F) undergoing SRT	No control group	YGTSS	28-point reduction in total YGTSS at 6 months (≈35%), sustained 24-point reduction at 12 months (≈29%); greater improvement in motor than vocal tics
Nagai et al. [20]	2014	Randomized controlled trial	11 TS pts receiving active-biofeedback	10 TS patients receiving sham-biofeedback	Tic frequency & subjective well-being indices	Both groups improved tic frequency and well-being, but no difference between active and sham; no demonstrable biofeedback skill learned.
Bloch et al. [21]	2016	Prospective open-label feasibility study	12 treatment-resistant adult TS pts (50% male), receiving deep rTMS to bilateral SMA	No control group.	YGTSS	Significant tic and OCD symptom improvement in TS + OCD subgroup only; no effect in TS-only group.

F, female; M, male, OCD; obsessive-compulsive disorder; pts, patients; Ref., reference; rTMS, repetitive transcranial magnetic stimulation; SMA, supplementary motor areas; SRT, stereotactic radiofrequency thermocoagulation; TS, Tourette syndrome; YGTSS, Yale Global Tic Severity Scale.

## Data Availability

No new data were created or analyzed in this study. Data sharing is not applicable to this article.

## Supplementary Materials

The following supporting information can be downloaded at: https://www.mdpi.com/article/10.3390/jcm15093393/s1, Table S1: Detailed search strategy for each database; Table S2: Risk-of-bias assessment of randomized controlled trials using RoB 2; Table S3: Risk-of-bias assessment of non-randomized studies and case reports using JBI tools. Table S4: PRISMA 2020 Checklist.

## Abbreviations

The following abbreviations are used in this manuscript:

TS	Tourette syndrome
PRISMA	Preferred Reporting Items for Systematic Reviews and Meta-Analyses
ESSTS	European Society for the Study of Tourette Syndrome
OCD	Obsessive-compulsive disorder
ADHD	Attention-deficit/hyperactivity disorder
TMS	Transcranial magnetic stimulation
ECT	Electroconvulsive therapy
DBS	Deep-brain stimulation
n	Number
THC	Δ9-Tetrahydrocannabinol
CBD	THC/cannabidiol
PEA	Palmitoylethanolamide
YGTSS-TTS	Yale Global Tic Severity Scale-Total Tic Score
GTS-QOL	Gilles de la Tourette Syndrome–Quality of Life Scale
PUTS	Premonitory Urge for Tics Scale
Hz	Hertz
CB1	Cannabinoid receptor type 1
