# Supplementary material for: Alternative Treatment of the Resistant-to-Treatment Tourette Syndrome—A Systematic Review
Source: J Clin Med. 2026 Apr 29;15(9):3393. doi: 10.3390/jcm15093393 (PMC13163666; doi:10.3390/jcm15093393)
Supplement: Supplementary file 1 [file jcm-15-03393-s001.zip › jcm-4254765-Table S1.pdf]

Supplementary Table S1. Detailed search strategy used in the systematic review.

| Database | Search query | Filters applied |
|---|---|---|
| PubMed | ("tourette syndrome") AND ("refractory to drugs" OR "refractory to medications" OR "refractory to treatment" OR "treatment resistant" OR "drug resistant" OR "medication resistant") AND ("alternative treatment" OR "alternative therapy" OR "therapy" OR "treatment") NOT ("deep brain stimulation") | Publication date: from 2010 onward |
| Embase | ('tourette syndrome') AND ('refractory to drugs' OR 'refractory to medications' OR 'refractory to treatment' OR 'treatment resistant' OR 'drug resistant' OR 'medication resistant') AND ('alternative treatment' OR 'alternative therapy' OR 'therapy' OR 'treatment') NOT ('deep brain stimulation') | Publication date: from 2010 onward |
| Scopus | (TITLE-ABS-KEY("tourette syndrome")) AND (TITLE-ABS-KEY("refractory to drugs" OR "refractory to medications" OR "refractory to treatment" OR "treatment resistant" OR "drug resistant" OR "medication resistant")) AND (TITLE-ABS-KEY("alternative treatment" OR "alternative therapy" OR "therapy" OR "treatment")) AND NOT (TITLE-ABS-KEY("deep brain stimulation")) | Publication date: from 2010 onward |
