# Supplementary material for: Alternative Treatment of the Resistant-to-Treatment Tourette Syndrome—A Systematic Review
Source: J Clin Med. 2026 Apr 29;15(9):3393. doi: 10.3390/jcm15093393 (PMC13163666; doi:10.3390/jcm15093393)
Supplement: Supplementary file 1 [file jcm-15-03393-s001.zip › jcm-4254765-Table S2.pdf]

Supplementary Table S2. Risk of bias assessment of randomized controlled trials using the RoB 2 tool.

**Study (Author, Year)**	**Randomization Process**	**Deviations from Intended Interventions**	**Missing Outcome Data**	**Measurement of Outcome**	**Selection of Reported Result**	**Overall Risk of Bias**
Müller-Vahl et al., 2023	Low	Low	Some concerns	Some concerns	Low	Some concerns
Nagai et al., 2014	Some concerns	Low	High	Some concerns	Low	High
