# Supplementary material for: Alternative Treatment of the Resistant-to-Treatment Tourette Syndrome—A Systematic Review
Source: J Clin Med. 2026 Apr 29;15(9):3393. doi: 10.3390/jcm15093393 (PMC13163666; doi:10.3390/jcm15093393)
Supplement: Supplementary file 1 [file jcm-15-03393-s001.zip › jcm-4254765-Table S3.pdf]

Supplementary Table S3. Risk of bias assessment of non-randomized studies, case series, and case reports using JBI critical appraisal tools.

Study (Author, Year)	Study Design	Clear Inclusion	Exposure Measured	Outcome Measured	Follow-up Adequate	Confounding Addressed	Overall Risk of Bias
Bloch et al., 2021	Uncontrolled interventional study	Yes	Yes	Yes	Some concerns	No	High
Bloch et al., 2016	Open-label study	Yes	Yes	Yes	Some concerns	No	High
Li et al., 2020	Case series	Some concerns	Yes	Yes	Some concerns	No	High
Hasan et al., 2010	Case report	Yes	Yes	Yes	N/A	No	High
Jakubovski et al., 2017	Case report	Some concerns	Yes	Yes	N/A	No	High
Pichler et al., 2019	Case report	Yes	Yes	Yes	N/A	No	High
Kanaan et al., 2017	Case report	Yes	Yes	Yes	N/A	No	High
Trainor et al., 2016	Case report	Yes	Yes	Yes	N/A	No	High
Ye & Lippmann, 2014	Case report	Yes	Yes	Yes	N/A	No	High
Dehning et al., 2011	Case report	Yes	Yes	Yes	N/A	No	High
Zhuo & Li, 2014	Case report	Some concerns	Yes	Yes	N/A	No	High

N/A, not applicable
